# Bacteriological Quality of Foods and Water Sold by Vendors and in Restaurants in Nsukka, Enugu State, Nigeria: A Comparative Study of Three Microbiological Methods

**DOI:** 10.3329/jhpn.v29i6.9892

**Published:** 2011-12

**Authors:** Chukwuemeka K. Nkere, Nnenne I. Ibe, Christian U. Iroegbu

**Affiliations:** ^1^Biotechnology Programme, National Root Crops Research Institute, Umudike, PMB 7006, Umuahia, Abia State, Nigeria; ^2^Department of Microbiology, University of Nigeria, Nsukka, Enugu State, Nigeria

**Keywords:** Coliform, Comparative studies, *Escherichia coli*, Food contamination, Food  hygiene, *Klebsiella pneumoniae*, Water pollution, Nigeria

## Abstract

Bacterial count in prepared food or water is a key factor in assessing the quality and safety of food. It also reveals the level of hygiene adopted by food handlers in the course of preparation of such foods. This comparative study evaluated the bacteriological quality of food and water consumed in Nsukka, Enugu state, Nigeria, using three bacteria enumeration methods. Data obtained are assumed to reflect the level of personal and environmental hygiene in the study population. Ten types of foods—beans, *yam, abacha, okpa, moimoi,* pear, cassava *foofoo*, rice, *agidi,* and *garri*—and 10 water samples were evaluated for bacteriological quality, precisely determining the level of coliform contamination, using the most probable number (MPN), lactose fermentation count (LFC), and *Escherichia coli* count (ECC) methods. Bacterial counts differed significantly (p<0.05) among the various food samples. However, this did not differ significantly in the three methods used for the enumeration of coliforms, suggesting that any of the three methods could be validly used for such studies with confidence. *Escherichia coli* and *Klebsiella pneumoniae* were the two major coliforms identified among 98 coliform isolates obtained from the various food samples, of which 78 (79.6%) were assumed to be of human origin on account of their ability to grow at 44 °C. The level of coliform contamination in the food samples from vendors and restaurants (geometric mean count 7.64-9.21; MPN ≥50) were above the accepted 10^4^ colony-forming unit/g or MPN ≤10 limits. The results of the study, therefore, call for stringent supervision and implementation of food-safety practices and regular education on food and personal hygiene among food vendors.

## INTRODUCTION

Food and water in particular have been described as vehicles for the transmission of microbial diseases, among which are those caused by coliforms (1). Coliforms are Gram-negative, rod-shaped, non-spore-forming aerobes and facultative anaerobes that ferment lactose to produce acid and gas within 48 hours at 35 °C. They are generally recognized as the normal flora of the intestine of humans and animals, although some coliforms, including *Salmonellae, Shigellae,* and enteropathogenic *Escherichia coli*, are notable enteric pathogens. The presence of coliforms in food and water would, therefore, generally connote faecal contamination, resulting in the risk of exposure to pathogens that cause gastrointestinal diseases, such as diarrhoea and typhoid fever. Poor environmental sanitation is largely responsible for much of the contamination, and poor personal hygiene, particularly among food handlers, accounts specifically for the contamination of foods while improper storage leads to multiplication of pathogens in food to infective doses. In the resource-poor tropical countries of the world, particularly in sub-Saharan Africa, foods are often preserved at ambient temperatures long before consumption, improperly handled by food vendors, and sold in streets in the dirty unhygienic environment (2-6). Most vendors have limited education and, therefore, lack knowledge on proper handling of the food and on the effects of improper handling with reference to transmission of food-borne pathogens.

The conditions described above prevail in Nsukka, a rural town in Enugu state in south-eastern Nigeria, and are compounded by inadequate access to good pipe-borne water, poor drainage systems, and lack of appopriate waste-disposal facilities. This lack of social amenities and municipal utilities has, in no small measure, contributed negatively to poor personal and environmental hygiene of food vendors in this locality. It is with this background that this investigation on the bacteriological quality of foods sold by vendors and in restaurants in Nsukka town is reported.

## MATERIALS AND METHODS

### Collection of samples

In total, 76 samples—66 of various foods and 10 of water—were examined. The foods consisted of nine samples each of beans and yam and six each of *abacha, moimoi, okpa,* rice, *agidi, garri,* cassava *foofoo,* and pear collected in sterile universal containers (Sterilin) from street food vendors and from restaurants. Each sample was properly identified with a number code, subject name, type of food, and state of the food (fresh or reheated). Samples were sent to the laboratory within two hours after collection in a cold-box containing ice-blocks. The constituent recipe and nature of each food are presented in Table 1.

### Bacteriological analysis

Three methods—lactose fermentation count (LFC), *E. coli* count (ECC), and the most probable number (MPN) or broadly two techniques (plate count and MPN)—were used simultaneously to estimate the coliform load in each food and/or water sample. For the plate count technique, approximately 2 g of food was homogenized in sterile saline, and the volume of the homogenate was made up to 20 mL to obtain a 1:10 suspension. The suspension was further serially diluted 10-fold down to 10^-5^. In the case of the LFC determination, a 0.1-mL inoculum was taken from the 10^-5^ dilution, placed onto duplicate MacConkey agar (MCA) plates and evenly spread over the agar surface using a sterile glass-rod spreader. One of the duplicate plates was incubated at 37 °C and the other at 44 °C for 24 hours. The ECC followed the same protocol as the LFC above, except that inoculum was taken from the 10^-4^ dilution instead of 10^-5^ and eosin methylene blue agar (EMBA) plates were inoculated instead of MCA. After 24 hours, the plates were examined for growth, and the colonies were counted. Discrete colonies were randomly selected from each plate (from both temperatures of incubation) and were characterized using standard bacteriological techniques to ascertain that they were coliforms and confirm the specific identity of the isolates (7).

For the MPN estimation, 20 g of the food sample was mechanically ground to coarse particles and washed in 200 mL of sterile saline by centrifugation. A 50-mL volume of the washing (supernatant) was added to 50 mL of double-strength MacConkey broth containing bromocresol purple; then 10-mL volume to 10 mL of double-strength MacConkey broth in a second tube; and 1-5 mL of single strength MacConkey broth in the third tube, each fitted with a Durham tube. The experiment was observed for colour change from purple to yellow and gas production for up to 48 hours at 37 °C. The MPN value was read from McCrady's Table (8,9). The water samples were bacteriologically examined using the same methods described above.

### Statistical analysis

Analysis of variance (ANOVA), using the SPSS software (version 10.0), Fisher's least significant difference (F-LSD), and Student's *t*-test statistics, was used for comparing the geometric means of bacterial counts (10).

## RESULTS

Two broad techniques—the MPN and the plate count (LFC and ECC)—were applied in the investigation. The pink colonies of the lactose fermenters on MCA and the *E. coli* colonies with green metallic sheen on EMBA were counted visually, and the counts taken together would constitute the total coliform count. Most food samples were heavily contaminated. The geometric mean count (GMC) in the foods for the total count assumed from the LFC ranged from 7.64 to 9.21 while that of ECC ranged from 6.34 to 7.81 (Table 2).

The proportion of the various types of contaminated food sometimes varied according to the technique used in the estimation of coliform load or count. For example, in LFC, 77.8% of the bean samples were contaminated while the ECC gave 22.2% contamination for the same food sample. However, for some types of food, the proportion contaminated was the same for different enumeration techniques. For example, in both LFC and ECC, 66.7% of *moimoi* and 50% of rice were contaminated (Table 2). The MPN count ranged from 0 to 180 (Table 3). A comparison of the three methods of isolation using the percentage of positive samples (Table 3 and 4) showed no significant difference between them (p>0.5). However, coliform count obtained for the various types of food differed significantly (p<0.05). Table 5 and 6 compared the GMC (LFC and ECC) and MPN, respectively, of food taken immediately after cooking and food taken from plates when served soon or ≥2 hours after cooking. The data showed a significant (p<0.05) difference between the coliform count in freshly-cooked food and the same food when later served.

**Table 1.** Description of foods analyzed

Food	Description	Form presented	Method of preparation
*Yam (Discorea esculanta)*	*Yam* porridge	Cooked	The bark is peeled, cut into small cubes, and cooked into porridge with fresh palm oil, crayfish, onions, vegetable, and spices, or served as cooked slices with spiced, salted fresh palm oil
*Abacha*	Cassava tapioca porridge	Cooked but served as salad with uncooked vegetables and palm oil	The bark is peeled, cooked till done, and sliced intopieces. The slices are steeped into clean water overnight before washing thrice in clean running water. This is then mixed with red palm oil, vegetables, fruits, and dried fish
*Okpa*	*Bambara* nut pudding	Cooked	*Bambara* nut-flour is measured out; red palm oil is rubbed into the flour until it appears homogenously yellow; warm water is added sequentially with stirring until it becomes a slurry. The slurry is dispensed in 100-200 mL into small heat-resistant polythene bags which are then lowered into boiling water to cook
*Moimoi*	*Cowpea* (bean) pudding	Cooked	Cowpea is soaked for 1-2 hours, dehusked, and wet-milled. Palm oil, condiments, onions, spices, dried fish, and fresh palm oil are added. These are mixed with water gradually until it becomes a homogenous slurry. The slurry is dispensed as described for *okpa* above and cooked into pudding in boiling water
Pear	*Avocado* pear	Fresh uncooked	Not applicable
Cassava *foofoo*	Cooked Cassava dough (non-baked bread-like dough)	Cooked andserved with vegetable soup containing meat and/or fish	Cassava is peeled and steeped in clean water for 3-4 days to ret. The retted cassava is sieved into a muslin bag, and water is pressed out to obtain a paste. A desired quantity of the paste is reconstituted into slurry in a frying-pot and cooked with constant stirring until it gels into firm bread-like dough. It is molded into round balls and served with vegetable soup as earlier indicated
Rice	Stewed-rice or *jollof*	Cooked	Rice may be cooked white and served with stew or cooked with all ingredients, including fish or meat, crayfish, palm oil, onions, tomatoes, and spices added as *jollof* rice
*Agidi*	Corn starch pudding	Cooked	Wholesome corn grains are either soaked in clean water for two days or boiled for not more than 20 minutes before cooling and leaving to soak overnight. The soaked corn is then wet-milled, mixed with water, and sieved through clean muslin cloth into a basin of clean water. The debris on the cloth are discarded, and the contents of the water in the basin are allowed to settle. The clear supernatant is removed, and the solid sediment is reconstituted into slurry with the residual water. The slurry is cooked with constant stirring until it forms a soft gel. It is then put into desired containers to cool and served with biscuit bone pepper-soup

In total, 98 coliform strains were isolated and identified as *Klebsiella pneumoniae* (45%), *E. coli* (51%), and *Enterobacter* (4%). Of the 98 isolates, 78 (79.6%), made up of 37 (47.4%) *K. pneumoniae* and 41 (52.6%) *E. coli* strains, showed growth at 44 °C while the remaining 20 (20.4%), including all the strains identified as *Enterobacter*, did not grow at 44 °C (Table 7).

**Table 2.** LFC and ECC of bacteria in food samples

**Type of food**	**No. of samples examined**	**No. of +ve samples (%)**		GMC (Log10 CFU/mL or g)*	
		**LFC**	**ECC**	**LFC**	**ECC**
**Beans**	**9**	**7 (77.8)**	**2 (22.2)**	8.41	6.46
***Yam***	**9**	**6 (66.7)**	**4 (44.4)**	8.35	6.93
***Abacha***	**6**	**5 (83.3)**	**5 (83.3)**	9.26	7.81
***Okpa***	**6**	**4 (66.7)**	**3 (50)**	8.87	7.48
***Moimoi***	**6**	**4 (66.7)**	**4 (66.7)**	8.65	7.41
**Pear**	**6**	**6 (100)**	**6 (100)**	9.21	6.34
**Cassava (*foofoo*)**	**6**	**2 (33.3)**	**1 (16.7)**	7.64	6.50
**Rice**	**6**	**3 (50)**	**3 (50)**	8.75	6.54
***Agidi***	**6**	**6 (100)**	**5 (83.3)**	8.64	7.40
***Garri***	**6**	**4 (66.7)**	**4 (66.7)**	8.65	7.53
**Water**	**10**	**8 (80)**	**8 (80)**	5.32	4.46

*Except for the water samples in which the bacterial counts are expressed as CFU/mL, all others are expressed as CFU/g;

CFU=Colony-forming unit;

ECC=*Escherichia coli* count;

GMC=Geometric mean count;

LFC=Lactose fermentation count;

+ve=Positive

**Table 3.** MPN count of bacteria in food samples

**Type of food**	**No. of stamples examined**	**No. positive/most probable number (MPN/g or mL) range†**		
		**0-10**	**11–50**	**51-180**
**Beans**	**9**	**3**	**-**	**6**
***Yam***	**9**	**3**	**1**	**5**
***Abacha***	**6**	**-**	**-**	**6**
***Okpa***	**6**	**2**	**1**	**3**
***Moimoi***	**6**	**2**	**-**	**4**
**Pear**	**6**	**-**	**-**	**6**
**Cassava (*foofoo*)**	**6**	**4**	**1**	**1**
**Rice**	**6**	**3**	**-**	**3**
***Agidi***	**6**	**2**	**-**	**4**
***Garri***	**6**	**3**	**-**	**3**
**Water**	**10**	**2**	**-**	**8**


†Except for the water samples in which the bacterial counts are expressed as MPN/mL, all others are expressed as MPN/g;

MPN=Most probable number

## DISCUSSION

The study was designed to determine the bacteriological quality of foods and water sold and consumed in Nsukka town. The main aim was to use this means to evaluate the level of personal and environmental hygiene in the area. All the food samples examined were obtained ready for consumption from vendors and stewards in restaurants. The water samples collected, along with the food samples, were those presented for drinking or used in washing utensils for serving the foods. The contaminated water samples could be the direct sources of ingestion of enteric pathogens or they could introduce pathogens to the foods through serving plates when they are used for washing.

Coliforms in general and *E. coli* in particular are conventionally referred to as indicators of faecal contamination. The presence of coliform in the food and water samples in this study was inferred from colony morphology in the selective and differential media used. The main selective factor in the media was inclusion of bile salts, which, to a large extent, inhibit the growth of Gram-positive organisms and other non-enteric bacteria. Random selection and characterization of colonies from the plates determined the presence of specific bacteria and confirmed that the study essentially enumerated coliforms.

**Table 4.** Percentage of positive food samples in different enumeration methods

**Type of food**	**Percent distribution of coliform-positive food samples according to enumeration methods**		
	**MPN**	**LFC**	**ECC**
**Beans**	**67.2**	**77.8**	**22.2**
***Yam***	**57**	**66.7**	**44.4**
***Abacha***	**92**	**83.3**	**83.3**
***Okpa***	**42**	**66.7**	**350**
***Moimoi***	**67**	**66.7**	**66.7**
**Pear**	**96.3**	**100**	**100**
**Cassava (*foofoo*)**	**20**	**33.3**	**16.7**
**Rice**	**48.1**	**50**	**50**
***Agidi***	**67**	**100**	**83.3**
***Garri***	**50**	**66.7**	**50**
**Water**	**92.4**	**80**	**80**


ECC=*Eschericia coli* count;

LFC=Lactose fermentation count;

MPN=Most probable number

**Table 5.** Bacterial count according to condition of food

**Food condition**	**GMC (Log10 CFU/mL or g)**	
	**LFC**	**ECC**
**Freshly-prepared‡/cooked**	**7.89**	**6.77**
**Served in plates**	**8.72**	**7.50**

‡This includes those foods (e.g. *abacha* and pear) prepared without heating;

CFU=Colony-forming unit;

ECC=*Escherichia coli* count;

GMC=Geometric mean count;

LFC=Lactose fermentation count

Technically, the coliform-enumeration methods used fall into two groups: MPN and plate count. The ECC and LFC are plate count methods. The ECC method is specifically used for enumerating *E. coli* while the LFC captures the total coliform count because all coliforms, with the exception of strains that have mutated, are lactose fermenters. The results obtained from the MPN and LFC showed a higher degree of agreement, and this would be expected since both the techniques gave the total count of the coliforms present. The LFC and ECC values for certain food samples varied as would also be expected given that *E. coli* is only one of the various coliforms counted in the LFC method. In a few cases, however, the values obtained with both plate count methods (LFC and ECC) were similar, and in such cases, *E. coli* was invariably the predominant organism isolated. However, there was no significant difference (p>0.05) among the three enumeration methods, which implies that any of these enumeration methods could be used for determining the level of coliform contamination. However, each method has its specific use. Thedetection of *E. coli*, as mentioned earlier, indicates faecal contamination. Also, certain strains of *E. coli* have been shown to be enteropathogenic (1,11), and hence the importance of inclusion of ECC as one of the enumeration methods. MPN, on the other hand, is generally not considered definitive as the plate count methods, which enumerate colonies of living organisms; the MPN values are only estimates. MPN is particularly useful in estimating coliform counts in materials, such as milk, food, water, and soil, with contaminations of <100 colony-forming unit (CFU)/g (12). Applying the three different techniques would ensure a more accurate determination and greater chances of detecting false positives and false negatives.

**Table 6.** Percentage of coliform-positive samples in relation to condition of food using the MPN count method*

**Type of food**	**Freshly-prepared/cooked**	**Served in plates**
**Beans**	**3.9**	**100**
***Yam***	**10**	**100**
***Abacha***	**100**	**100**
***Okpa***	**0**	**10**
***Moimoi***	**0**	**100**
**Pear**	**88.9**	**100**
**Cassava (*foofoo*)**	**0**	**1.7**
**Rice**	**0**	**100**
***Agidi***	**0**	**100**
***Garri***	**0**	**0**
**% frequency of positive samples**	**40**	**90**

*All food samples were kept under ambient temperature (room temperature);

MPN=Most probable number

The coliform counts obtained in the foods and water samples in this study are far higher than the 10^4^ CFU/g taken as tolerable in foods in developed countries (13), which implies that foods and water sold in the town are generally unhygienic. The *E. coli* and *K. pneumoniae* isolates that showed growth at 44 °C reflect the human origin of the contaminants (7-9). The presence of coliforms of presumably human origin points to the risk of exposure to enteropathogens, including *Salmonella, Shigella,* and enteropathogenic *E. coli*, which were not specifically investigated in this study. The high level of food contamination observed had also been reported earlier in Nigeria and from other developing countries, including Senegal, Bangladesh, and Ghana (1,11,14-16). What these regions have in common are poverty and low standard of personal and environmental hygiene.

**Table 7.** Bacterial isolates from samples

Biochemical tests											Isolate	Frequency	Human origin No. (%)	Other No. (%)
Gram stain	Lactose	Mannose	Glucose	Maltose	Sucrose	Oxidase	Citrate	Indole	Urea	H2S				
− rods	AG	AG	AG	AG	AG	−	+	−	+	**−**	***Klebsiella pneumoniae***	**44**	**37 (84.1)**	7 (15.9)
− rods	AG	AG	AG	AG	AG	−	−	+	−	**−**	***Escherichiacoli***	**50**	**41 (82.0)**	9 (18.0)
− rods	AG	AG	AG	AG	AG						***Enterobacter***	**4**	**0 (0.0)**	4 (100)
Total												**98 (100)**	**78 (79.6)**	20 (20.4)

AG=Acid and gas;

+=Positive; −=Negative

The suggested bacteriological standard for drinking-water from unchlorinated water, mostly drawn from hand-pumps, commonly used in rural communities and other sources (also adapted for food samples), is the MPN value of 0, which is rated excellent and desirable; values between 1 and 10 are still acceptable but values of ≥50 signify gross pollution or contamination and, therefore, not fit for human consumption (7,8). The plate counts also indicated unacceptably high levels of contamination of the food samples.

Coliforms and intestinal pathogens in general are easily destroyed by pasteurization (at ∼70 °C for 30 minutes), and this may explain why pear and *abacha*, which require no heat for preparation, had relatively higher coliform counts. The heat-processed food samples collected immediately after cooking and directly from the cooking-pot showed little or no coliform contamination but when collected as served foods sometime later, these showed high contamination (Table 6). The latter observed contamination presumably originated from water used in washing the utensils and plates or directly from the hands or bodies of vendors or the restaurant stewards. Of course, once introduced in the much cooled food samples and left at ambient temperatures for a while, the contaminating coliforms would multiply to higher counts in foods (14).

High concentrations of coliforms in foods have been associated with such symptoms as nausea, vomiting, retching, abdominal cramp, diarrhoea, and prostration (17,18); thus, the mere presence of coliforms at high concentrations even without the concomitant presence of well-known enteropathogens could be the cause of much morbidity among the study population. The non-availability of good water supply to most families in Nsukka, sometimes constraining them to buy from water-tankers of unsure sources or even to collect from broken pipes and gutters, would make the hygiene situation in the homes even more precarious. Given the inadequacy of the public water supply, most houses with installed water closets cannot use these, and others do not have good toilet facilities. Consequently, many people defaecate in nearby bushes or in the gutters at night, thereby contaminating the environment, especially during floods. This is ultimately reflected in the high coliform counts and predominance of coliforms of human origin obtained in the present study.

### Conclusions

The situation calls for strict public-health regulations regarding the sale of foods on streets by vendors and/or in restaurants, regular laboratory checking of foods and water from these sources for microbiological quality, and routine inspection by health inspectors of foods from vendors and restaurants and the environment where they are prepared and sold. The ultimate solution remains with provision of adequate portable water, teaching of good-hygiene practices, including handwashing, sometimes during meetings of market unions and communities, and enforceable legislation on food-handling and environmental sanitation may improve the situation. These would, however, go a long way to reduce the prevailing morbidity due to gastroenteritis in the study population.
